# Site Suitability and Air Pollution Impacts of Composting Infrastructure for California’s Organic Waste Diversion Law

**DOI:** 10.1021/acs.est.4c06371

**Published:** 2024-10-30

**Authors:** Brendan P. Harrison, Wilson H. McNeil, Tao Dai, J. Elliott Campbell, Corinne D. Scown

**Affiliations:** † Energy and Biosciences Institute, University of California, Berkeley, Berkeley, California 94720, United States; ‡ Energy Technologies Area, Lawrence Berkeley National Laboratory, Berkeley, California 94720, United States; § Department of Civil and Environmental Engineering, University of California, Berkeley, Berkeley, California 94720, United States; ∥ Biosciences Area, Lawrence Berkeley National Laboratory, Berkeley, California 94720, United States; ⊥ Life-Cycle, Economics and Agronomy Division, Joint BioEnergy Institute, Emeryville, California 94608, United States; # Environmental Studies Department, University of California, Santa Cruz, Santa Cruz, California 95064, United States

**Keywords:** composting, landfills, methane, air pollution, environmental justice

## Abstract

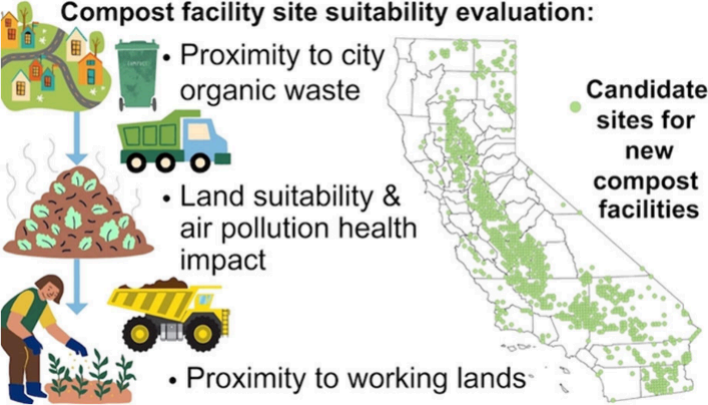

California’s organic waste diversion law, SB 1383, mandates a 75% reduction in organics disposal by 2025 to reduce landfill methane emissions. Composting will likely be the primary alternative to landfilling, and 75–100 new large-scale composting facilities must be sited in the state to meet its diversion goal. We developed a strategy for evaluating site suitability for commercial composting by incorporating land-use, economic, and environmental justice criteria. In our Baseline scenario, we identified 899 candidate sites, and nearly all are within a cost-effective hauling distance of cropland and rangelands for compost application. About half of sites, mostly in rural areas, are not within a cost-effective collection distance of enough municipal organics to supply an average-sized facility. Conversely, sites near cities have greater access to organics but cause greater health damages from ammonia and volatile organic compounds emitted during the composting process. The additional required composting capacity corresponds to $266–355 million in annual damages from air pollution. However, this excludes avoided emissions from landfilling, and damages could be reduced by 56% if aerated static piles are used instead of windrows. Siting a higher number of smaller decentralized facilities could also help equally distribute air pollution to avoid concentrating burdens in certain communities.

## Abstract 2

We identified and analyzed suitable compost facility sites in California to help the state meet its organic waste diversion law.

## Introduction

In the U.S., organic resources account for approximately 63% of disposed waste, and the anaerobic decomposition of this material in landfills produces over 17% of the country’s total anthropogenic methane (CH_4_) emissions.^1,2^ Diverting organic resources from landfills to reduce CH_4_ emissions is a powerful climate mitigation strategy, as CH_4_ is over 80 times more effective at warming the planet than carbon dioxide (CO_2_) over a 20 year period.^3−6^ Therefore, the rapid deployment of organic waste diversion, along with other strategies to cut CH_4_ emissions, could play a critical role in slowing near-term warming, as societies begin the long and difficult transition away from fossil fuels.^7^

The majority of municipal waste is still landfilled in the U.S., and waste diversion and emissions mitigation efforts are largely pursued at the state level. Currently, policies designed to limit the landfilling of organic waste have been adopted in 10 states, nine cities, and the District of Columbia, representing approximately 100 million people or about 30% of the population of the U.S.^9,10^ For example, California passed a landmark organic waste diversion law in 2016 to reduce CH_4_ emissions from landfills, which are one of the largest sources in the state and account for 20% of total statewide CH_4_ emissions.^3,8^ Its short-lived climate pollutant reduction law, SB 1383, requires a 75% reduction of organic waste landfill disposal from the 2014 level by 2025.^11^ While organic waste can be converted to useful products through a variety of strategies, aerobic composting, which emits substantially less CH_4_ emissions relative to landfilling, is expected to be the primary pathway to manage the state’s diverted organics due to its relatively low operational costs, high throughput capacity, and wide range of acceptable feedstocks.^3−5,12^ While anaerobic digestion will be used to treat a smaller portion of diverted organics, the resulting digestate is often still composted in order to convert the material into a valuable soil amendment.^13^ The application of compost to soils also offers many environmental and agronomic co-benefits.^14−17^

However, when accounting for the estimated expansion of composting capacity at existing facilities, California will still need an additional 75–100 facilities by 2025, equivalent to the number of existing composting facilities permitted to handle municipal organics, to process the approximately 5 million metric tonnes (t) of additional compostable materials that must be diverted from landfills annually.^12,18,19^ Siting new facilities can be a slow and difficult process, as compost companies face a variety of economic, regulatory, and logistical barriers.^12,19^ Because composting is a significant source of ammonia (NH_3_) and volatile organic compound (VOC) emissions, one of the greatest challenges in siting new facilities in California is complying with local air district regulations.^5,12,19,20^ Concerns about impacts to nearby communities from proposed sites may also be a barrier, especially in socioeconomically disadvantaged communities (DACs), where community members may have less capacity to cope with additional odor and air pollution and where waste facilities have been disproportionately sited.^12,19,21−23^ Composting facilities typically must also be located within a cost-effective hauling distance of agriculture to access markets and, at the same time, in proximity to population centers to reach municipal organic waste feedstock.^3,12,17,24^

Due to the complex and, at times, conflicting criteria for siting a compost facility, it remains unclear (1) whether there are enough suitable sites across the state for the 75–100 new facilities needed to meet SB 1383 and (2) whether there are regions of the state that are preferable for constructing new facilities. Here, we address these questions by conducting the first comprehensive site suitability and air pollution health impact analysis for new composting facilities in California, whose organic waste diversion policy, if successful, could serve as a model to other governments.^3^ In our analysis, we build upon Hall et al.’s recent cost and greenhouse gas optimization model for the regional expansion of composting capacity by identifying specific suitable sites for new facilities, estimating each site’s access to working lands and municipal organic waste, and quantifying the public health impact to surrounding communities from each facility’s air pollutant emissions.^25^ Our compost facility site suitability framework, based on land-use, economic, and environmental justice criteria, can also be used to help a growing number of other states, municipalities, and governments meet their organic waste diversion goals.^26^

## Methods

### Site Suitability Analysis

We identified suitable land in California for the siting of new composting facilities by applying several land suitability criteria. Land-use data came from the 2021 National Land Cover Database (NLCD), which classifies California’s land into eight land-use classes.^27^ From these, we include land classified as barren, shrub/scrub, grassland/herbaceous, and planted/cultivated, and we exclude land classified as open water, developed, forest, or wetlands. Developed areas were excluded because of the lack of available land area for large-scale composting operations, zoning restrictions, and potential public opposition to facilities arising from odor and public health concerns.^10,19^ Forests and wetlands were excluded, along with all protected land, due to the critical ecosystem services and habitat that they provide.^28−31^ To minimize potential nutrient runoff to waterways, we excluded a 30 m buffer area around open water and wetlands as well as land with a slope greater than 6%.^32−34^ Floodways and 100 year flood zones were also excluded to reduce the risk of damage to facilities and waterway contamination.^32,34,35^ In an alternative scenario, we excluded land located in SB 535 Disadvantaged Communities (DACs), which include communities with a high pollution burden and/or high socioeconomic vulnerability, as well as federally recognized tribes.^36^ This was done to explore the impact and potential trade-offs of avoiding siting facilities in vulnerable communities.^21,22,36^

After down-selecting suitable land for new composting facilities, we removed land parcels less than 23.4 hectares (ha), the average area of a compost facility in California.^37^ We then aggregated the remaining suitable land into specific sites for later analyses. This was done by first increasing the pixel size of the suitable land raster layer from 30 m × 30 m to 10 km × 10 km and then taking the centroid of each pixel to produce a point representing a candidate site for a new compost facility. Our site suitability analysis generated two sets of sites: our Baseline sites and our No-DAC sites, which are evaluated across a range of scenarios in later analyses (Table S1).

### Working Lands Proximity Analysis

Using the two sets of sites generated from our site suitability analysis, we conducted a working lands proximity analysis to estimate the area of working lands accessible to each composting facility for the eventual application of compost to soils. In previous work, we found that the distance that compost can be hauled to working lands is limited by economics, as hauling expenses make up the majority of the cost of compost to farmers and land managers.^3^ For our baseline Long Compost Transport scenario, we used a 160 km cost-effective hauling distance threshold, and we considered both cropland (cultivated land used to grow crops) and rangelands (land used to graze livestock), which are frequently referred to cumulatively as working lands.^3,24,38^ We excluded rangelands with a slope greater than 15%, as compost application to steep slopes may increase nutrient runoff and is a practice considered ineligible for state and federal programs that incentivize compost application.^39,40^ Road data were acquired from the U.S. Census Bureau’s TIGER/Line shapefiles.^41^

To estimate the area of working lands accessible to each site for compost distribution, we used the service area tool in QGIS, an open-source geographic information system program.^42^ This tool highlights the areas of a network that can be reached from a point given a distance threshold. We then used QGIS’s convex hull tool to create a minimum bounding geometry for each service area. Each site’s service area was then spatially joined with the working lands that intersect it, allowing us to estimate the total area of accessible working lands.

For our working lands proximity analysis, we tested a total of six unique scenarios. In addition to our baseline Long Compost Transport scenario, we tested a 50 km distance threshold (Short Compost Transport) to estimate the amount of working lands that could receive compost from local facilities at a potentially lower cost, due to shorter hauling distances.^3^ We also tested a cropland-only scenario (Crop-only), which assumes that compost is used only as a source of fertility to enhance food production. We ran each of these three scenarios for our two sets of sites to produce six scenarios (Table S1).

### Organic Waste Proximity Analysis

In addition to working lands, we estimated the amount of municipal organic waste within a cost-effective collection distance of each compost facility. We used Lawrence Berkeley National Lab’s Biositing Tool to extract annual compostable municipal organic waste generation data (food waste, paper, and yard trimmings) for each census tract in California, and we converted from dry mass to wet mass using the average moisture content of each waste type.^43−46^ We exclude other sources of organic waste, such as agricultural residues and manure, as this material is often managed through on-farm methods and is not typically landfilled and, therefore, represents only a small fraction of the organic waste that must be diverted from landfills to meet SB 1383.^19^ While uncontaminated paper products can be recycled, we included paper in this analysis because “compostable” paper represents the largest category of landfilled paper products and accounts for approximately 17% of the compostable material in California’s waste stream.^47^

Using the same methods discussed in the previous section, we created service areas for each facility to estimate the amount of accessible municipal organic waste. We assumed that new facilities can access organic waste streams through curbside organic waste collection services, which, as of 2022, are required for most jurisdictions in California through SB 1383.^18^ In our baseline Medium Feedstock Collection scenario, we used a 70 km cost-effective collection distance.^5,48^ We also considered high and low distance scenarios in our Long Feedstock Collection and Short Feedstock Collection scenarios, in which our baseline distance is doubled or halved, to test how collection distance influences organic waste access for facility sites across the state. We used our three collection distance scenarios for each of our two sets of sites, producing six scenarios (Table S1).

### Health Impact Analysis

The impact that a particular compost facility has on public health depends on factors such as regional air chemistry, population density, and composting method.^49,50^ While a significant body of literature exists on the health impacts of bioaerosol emissions on compost facility workers and those living very close to facilities, we focus on regional air pollutants, such as NH_3_ and VOCs, as they impact much larger populations.^51^ To investigate this, we estimated the public health impact from air pollutant emissions for each facility site using the InMAP source-receptor matrix (ISRM), a reduced-complexity air quality model that quantifies the health damages from primary and secondary fine particulate matter (PM_2.5_), which together account for 95% of premature deaths from air pollution.^50,52^ Using the ISRM, we predicted the change in the PM_2.5_ concentration surrounding each facility due to the emission of primary PM_2.5_ and PM_2.5_ precursors from the composting operation. The location-specific change in PM_2.5_ concentration is calculated based on existing air quality data and the additional air pollutants emitted during composting.^52^ Local population data are then used along with the change in PM_2.5_ concentration to quantify the additional annual air pollution-related mortalities using a concentration–response relationship.^53^ The annual mortality caused by air pollutant emissions from each facility is converted to monetary health damages using the U.S. Environmental Protection Agency (EPA)’s recommended value of a statistical life, which is $11.2 million in 2023 U.S. dollars (USD).^54^

We used municipal organic waste (food, yard, and organic fraction municipal solid waste) composting emission factors for NH_3_ and VOCs from a recent meta-analysis of composting emission studies (Table S2).^49^ We assume that each composting facility processes 60 × 10^3^ t yr^–1^, which is the average throughput for California facilities.^12^ Trucking emissions from hauling compost to working lands and transporting organic waste to facilities are also quantified and assigned to their respective facilities, and we assume that the material is hauled by heavy-duty diesel trucks over the maximum collection and hauling distances from our baseline scenarios. We used diesel emission factors for heavy-duty trucks and assume that trucks transporting raw feedstock have a capacity of 12.7 t and trucks transporting compost have a capacity of 21.8 t; we do not account for empty miles traveled.^3,55^ The impact of composting method on health impact is compared in this analysis by testing windrow composting and aerated static pile (ASP) emission factors, for our two sets of sites, to produce four scenarios (Table S1).^49^

## Results and Discussion

### Site Suitability Analysis

We conducted a site suitability analysis for new composting facilities in California. In our Baseline scenario, we identified approximately 5.1 × 10^6^ ha of suitable land, or 12.6% of California’s total land area, for siting new composting facilities. Of the five suitable land-use classes (shrubland, grassland, cropland, pasture, and unvegetated/barren), cropland contributed the largest portion of suitable land area (2.5 × 10^6^ ha), while pasture provided the least (1.1 × 10^5^ ha) (Figure S1). Protected land and land with unsuitable slope, together, accounted for approximately 90% of the land area excluded from the five suitable land-use categories. In our No-DAC scenario, we found that 3.3 × 10^6^ ha of land was suitable for siting facilities, a 35% reduction in area relative to the baseline. Because California’s highly productive agricultural region, the San Joaquin Valley, is home to the majority of cropland and DACs in the state, 70% of the land area excluded in the No-DAC scenario was cropland. Despite the large amount of cropland in DACs, cropland was still the dominant suitable land-use class in the No-DAC scenario, accounting for over one-third of the total suitable land area.

We aggregated the suitable land into 899 and 605 individual candidate composting sites in our Baseline and No-DAC scenarios, respectively. From our analysis, we found that even when all land in DACs is excluded, there are still more than enough suitable sites to accommodate the 75–100 new facilities needed to meet SB 1383.^12^ Of the nine regions in California, the agriculture-rich geographic center of the state, the San Joaquin Valley, had the most suitable facility sites in both scenarios, with 355 and 114 in our Baseline and No-DAC scenarios, respectively (Figure S2). The Sacramento Valley (Northern Sacramento Valley and Greater Sacramento regions), which is north of the San Joaquin Valley and also a rural, agricultural region, also had many suitable sites with 119 and 118 in our Baseline and No-DAC scenarios, respectively. Both the San Joaquin Valley and the Sacramento Valley (together called the Central Valley) have an abundance of flat cropland and little protected or developed land, so in terms of land suitability, they may be ideal for siting new composting facilities. However, flood risk remains an important consideration in these flood-prone regions, especially as climate change is expected to increase the risk of severe floods in both watersheds.^56^

We show that cropland in California’s Central Valley could provide a substantial area of suitable land for siting new compost infrastructure in the state, but siting facilities on former cropland could reduce food production in this important agricultural region. However, due to worsening drought conditions, approximately 20–36 × 10^4^ ha of irrigated cropland in the San Joaquin Valley is expected to be fallowed by 2040 to comply with the state’s Sustainable Groundwater Management Act.^57^ While others have considered repurposing this fallowed cropland for renewable energy production, conservation, or public recreation, farmers could also sell or lease fallowed portions of their land to compost companies.^58^ If we assume an average compost facility footprint of 23.4 ha, California could site all 355 San Joaquin Valley sites identified in our Baseline scenario using only 2–4% of the cropland estimated to be fallowed in the region by 2040. While water is typically added to compost piles, consumption is low (0.02–0.33 m^3^ t^–1^ feedstock) relative to crop irrigation, and compost application to farmland can substantially increase soil water holding capacity, potentially reducing irrigation requirements.^15,59^

### Working Lands Proximity Analysis

**Figure 1:**
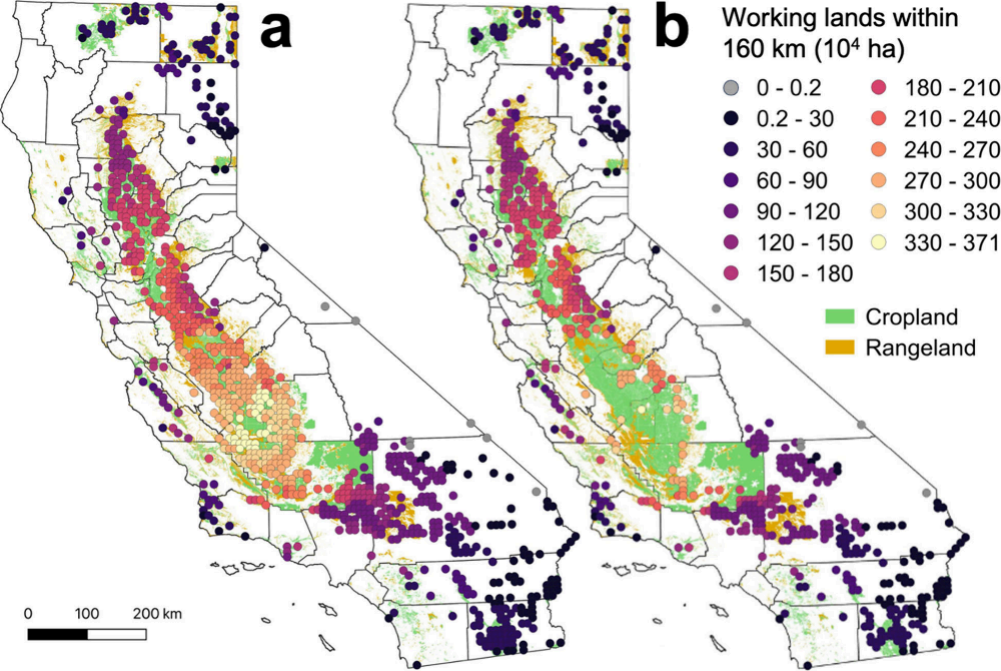
Area of working lands (10^4^ ha) accessible to each suitable compost facility site in the (a) Long Compost Transport and (b) No-DAC + Long Compost Transport scenarios. Gray points represent sites that cannot access a sufficient area of working lands to distribute their compost. Green polygons represent cropland, and light brown polygons represent rangelands. No-DAC is no disadvantaged communities.

We estimated the area of working lands within an economic distance threshold of each site to examine their ability to access agricultural markets. Unsurprisingly, San Joaquin Valley sites had the greatest access to working lands across all scenarios, averaging 259 × 10^4^ ha in the baseline Long Compost Transport scenario (Figure 1). However, over 80% of the sites within DACs are in this region; therefore, despite their proximity to working lands, sites in the San Joaquin Valley may face environmental justice concerns. While Sacramento Valley sites have access to less working lands on average (167 × 10^4^ ha in the Long Compost Transport scenario), siting in this region may be more desirable from an environmental justice perspective, as virtually no candidate sites are in DACs. In agreement with previous work, nearly all sites have access to sufficient working lands.^3^ If we assume an average facility throughput of 60 × 10^3^ t yr^–1^, a feedstock to compost conversion rate of 50% by mass, and a 13.5 t ha^–1^ compost application rate, then facilities need access to a minimum of 0.2 × 10^4^ ha of working lands to distribute their compost, a criterion that all but 7 out of 899 sites meet in the Long Compost Transport scenario.^12,39,60^ Importantly, this means that nearly all sites in urban and densely populated Southern California, which must process the massive amount of organics diverted from Los Angeles County landfills, do have access to sufficient working lands. However, this finding is likely unique to states with an abundance of farmland, such as California, as its expansive working lands are spread out across much of the state and interspersed with population centers, compared to, for example, New York, where state population is concentrated in New York City, far from the state’s primary agricultural regions; although, farmland in New Jersey and Pennsylvania could potentially be accessed (see additional discussion in the Supporting Information).

**Figure 2:**
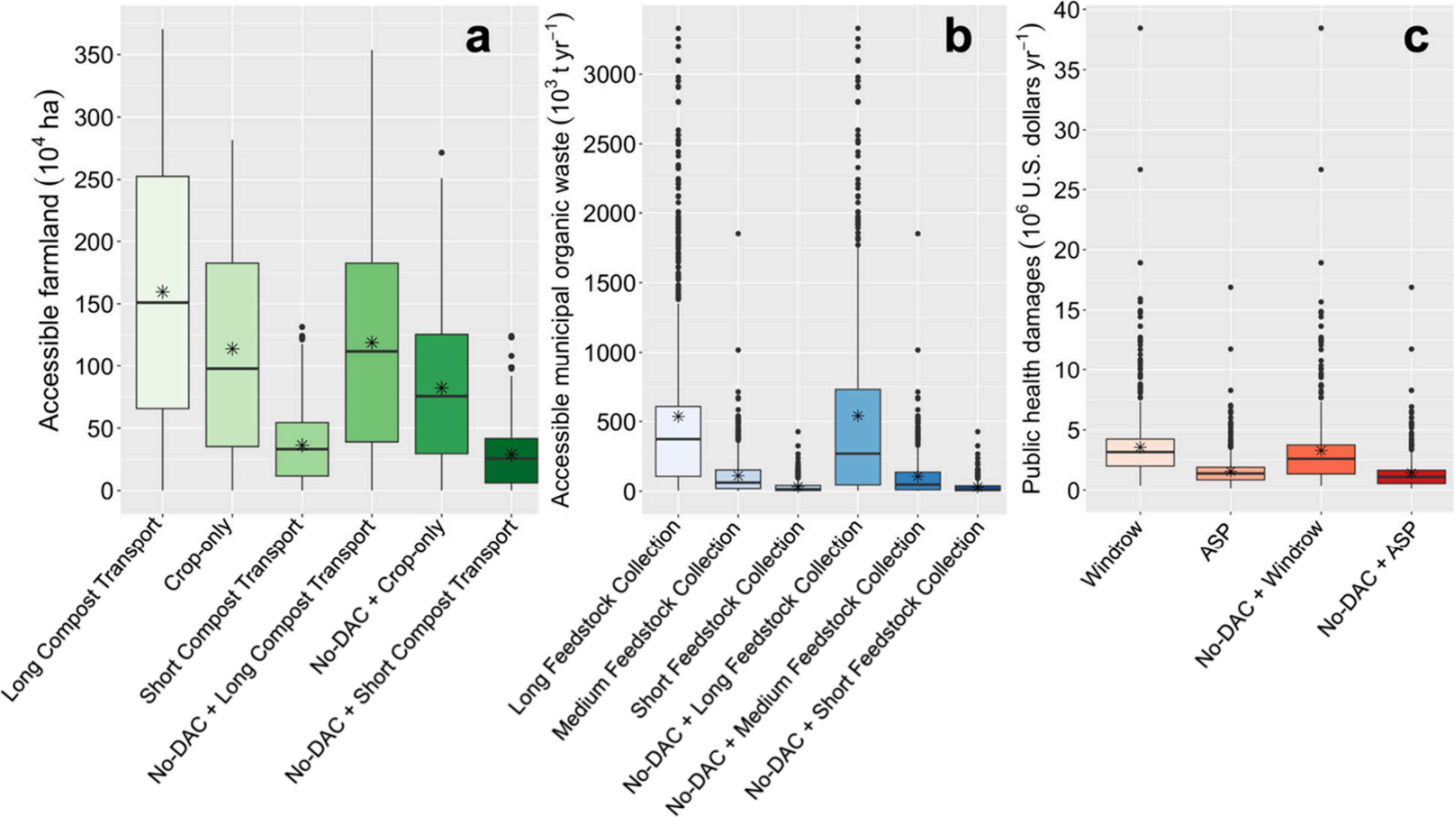
All scenario results for individual sites in the (a) working lands proximity analysis (in 10^4^ ha), (b) organic waste proximity analysis (in 10^3^ t yr^–1^), and (c) health impact analysis (in $10^6^ 2023 USD yr^–1^). The minimum area of working lands needed to distribute the compost from an averaged-sized facility is 0.2 ha, and the amount of organic waste feedstock needed for an average facility is 60 × 10^3^ t yr^–1^. The horizontal line represents the median of all candidate sites. The asterisk is the mean. The dots are outliers, and the whiskers depict the first and third quartiles. No-DAC is no disadvantaged communities, and ASP is aerated static piles.

For our Baseline sites, the average area of working lands accessible for the Long Compost Transport, Crop-only, and Short Compost Transport scenarios was 160 × 10^4^, 114 × 10^4^, and 36 × 10^4^ ha, respectively (Figures 1 and 2a; Figure S3). Because most sites in DACs were in the agriculture-rich San Joaquin Valley, the average area of working lands accessible to sites in the No-DAC scenarios was 19–28% less than the baseline scenarios, a potential trade-off of prioritizing environmental justice over access to agricultural markets. However, it is important to note that 99% of sites in the No-DAC + Long Compost Transport scenario still have access to sufficient working lands to distribute all compost produced, using the assumptions described previously.^12,39,60^ Even under the conservative Short Compost Transport scenarios, 88% and 90% of sites reach enough working lands in the No-DAC and Baseline scenarios, respectively (Figure S3).

While we use 160 km as our baseline hauling distance in this analysis, compost subsidies could potentially expand access to agricultural markets by helping farmers pay for transportation costs. In 2021, California’s Healthy Soils Program (HSP) distributed over $60 million through its Incentives Program to help farmers pay for compost, and the state will soon consider a compost tax credit fund that would allocate up to $120 million per year to help subsidize compost purchases.^61,62^ Using the economic model in the work by Hall et al., we estimate that a farmer who receives an HSP Incentive grant of $100,000 could cover the entire cost of applying 1900 t of compost to a 140 ha farm (the average farm size in California) while shipping their compost up to 320 km, twice the distance of our baseline value.^17,39,63^

Our analysis also highlights the importance of rangelands for compost distribution. In the Crop-only scenarios, which exclude rangelands, sites have access to 29–31% less working lands, on average, than in the baseline scenarios. While amending cropland soils with compost can improve crop yields and reduce dependence on synthetic fertilizers, compost application to rangelands has been shown to enhance carbon sequestration and improve plant production for grazing.^14,64^

Although agriculture is the primary market for compost and the focus of this analysis, other markets also exist and may soon expand. For example, cities are required under SB 1383 to procure 73 kg of diverted organic waste per capita in the form of an organic product, such as compost.^65^ In a previous analysis, we estimated that cities could apply 8–26% of SB 1383 diverted organic waste as compost to urban lands.^3^ Therefore, a significant opportunity exists to apply compost to city green spaces, such as community gardens and public parks, which could improve food security, sequester carbon, and reduce erosion.^66,67^

### Organic Waste Proximity Analysis

**Figure 3:**
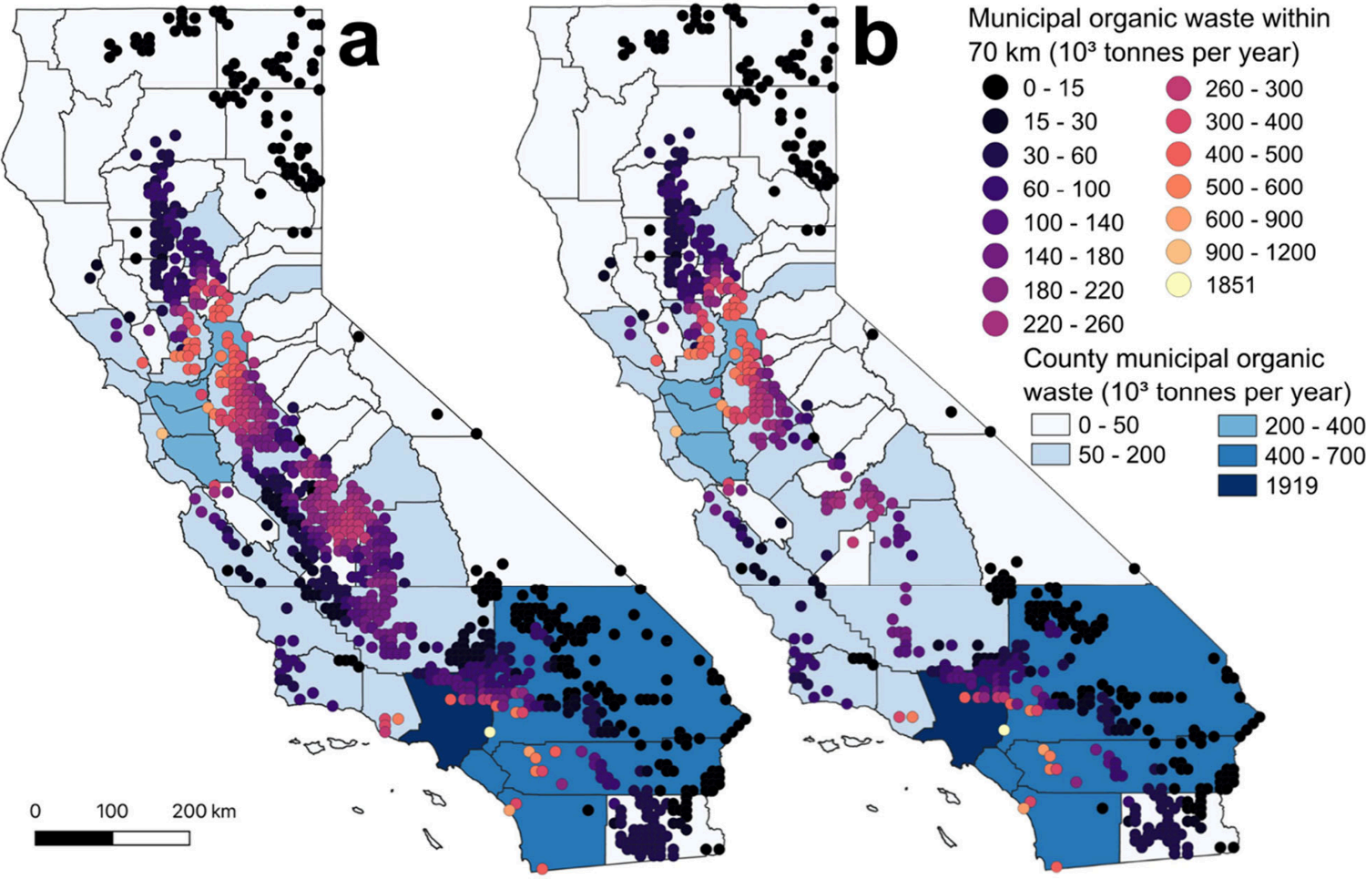
Amount of municipal organic waste (10^3^ t yr^–1^) accessible to each suitable compost facility site in the (a) Medium Feedstock Collection and (b) No-DAC + Medium Feedstock Collection scenarios. Counties are shaded by organic waste production with darker counties producing more waste. No-DAC is no disadvantaged communities.

We conducted an organic waste proximity analysis to estimate the amount of municipal organic resources accessible to each suitable site. When comparing regions within our baseline Medium Feedstock Collection scenario, sites in the urban Bay Area had access to the most organic waste on average, with 388 × 10^3^ t yr^–1^; however, this region had only 21 suitable sites, the fewest of any region (Figure 3). Sites in the Greater Sacramento and San Joaquin regions, in addition to having access to a large area of working lands, had the second and third most available organic waste, with 284 × 10^3^ and 145 × 10^3^ t yr^–1^ on average, respectively. While Southern California produces the most organic waste, sites in this region had access to an average of only 78 × 10^3^ t yr^–1^ of organic waste, as most facilities were sited in suburban or rural areas, far from the densely populated urban areas where most organic waste is produced. Although Northern California, which is rural and sparsely populated, had 77 suitable sites, each site had access to only 6 × 10^3^ t yr^–1^ on average, a mismatch in suitable land and organic resource availability.

When comparing across scenarios using our Baseline sites, facilities had access to, on average, 32 × 10^3^, 111 × 10^3^, and 536 × 10^3^ t yr^–1^ in our Short, Medium, and Long Feedstock Collection scenarios, respectively (Figures 2b and 3; Figure S4). No-DAC sites had similar averages to Baseline sites across the three scenarios. While our working lands analysis showed that nearly all sites had access to sufficient land to distribute the compost produced by an average facility, only about half of the sites in our baseline Medium Feedstock Collection scenario had access to at least 60 × 10^3^ t yr^–1^ of municipal organic waste. However, when collection distance is doubled in our Long Feedstock Collection scenario, sites could access approximately 80% more organic waste, on average, which allowed 73% of No-DAC and 80% of Baseline sites to access at least 60 × 10^3^ t yr^–1^ of feedstock. Longer collection distances may prove critical in accessing the large amount of organic waste produced deep within sprawling urban areas such as Los Angeles. This could be achieved with strategically sited waste transfer stations, which reduce collection costs by transferring waste from many small collection trucks to larger vehicles that can efficiently haul loads over long distances.^68^

Although we consider all municipal organics produced for this analysis, the fraction of organics actually available for composting is difficult to estimate and may vary substantially over space and time. This fraction depends on factors such as the public adoption of organic waste separation, the implementation of mandated curbside collection programs, hauling contracts, and the amount of contamination in the waste stream, all of which may change as infrastructure and public awareness grow.^18,19,69^ Facilities that cannot access enough municipal organic waste could supplement their operations with agricultural waste, but this material usually does not count toward SB 1383’s diversion goal and typically has lower tipping fees.^18^ However, accessing agricultural waste may be especially relevant to the many candidate sites that are located on cropland and are far from the organic waste produced in population centers.

While the average California compost facility throughput is 60 × 10^3^ t yr^–1^, smaller industrial-scale facilities may play an important role in regions that produce less organic waste, such as sparsely populated Northern California. Community-scale composting projects could also play a role in reducing the burden on California’s organics handling infrastructure, especially in urban areas far from sites suitable for large composting facilities.^25^ For example, if we assume an individual community site can process 0.25 × 10^3^ t yr^–1^, a network of community composting sites across Los Angeles County’s parks, schools, churches, and gardens could process all SB 1383 diverted organic waste, or the equivalent throughput of 25 average-sized composting facilities.^70−75^ Analyses of urban areas outside of California, such as New York City and Chicago, also show a high potential for decentralized composting systems.^70,76^ In addition to processing waste, community composting sites also provide educational opportunities, help develop a local circular economy, reduce transportation emissions, and support local food production in urban areas.^25,70,76,77^

### Health Impact Analysis

**Figure 4:**
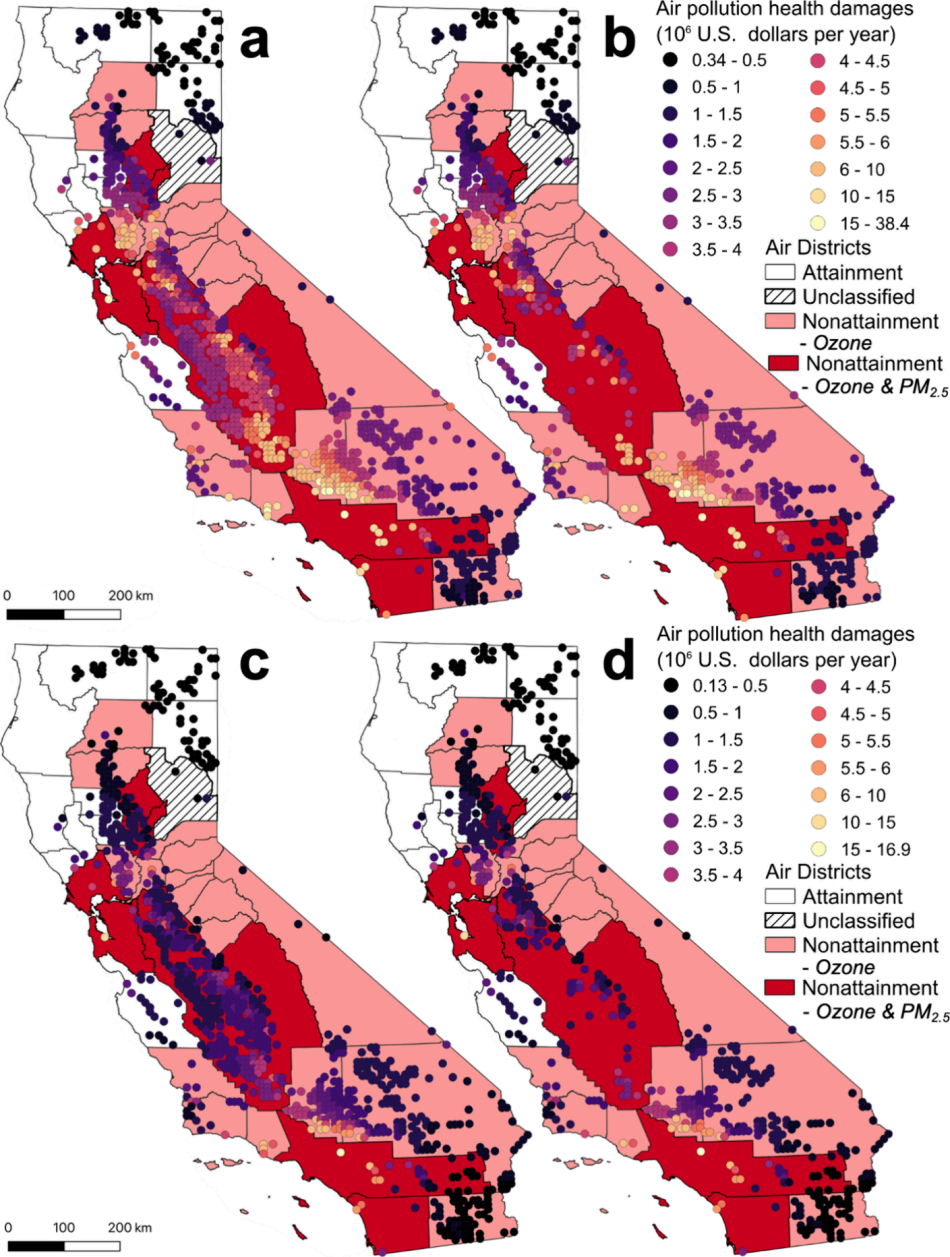
Air pollution health damages ($10^6^ 2023 USD yr^–1^) of each suitable compost facility in the (a) Windrow, (b) No-DAC + Windrow, (c) ASP, and (d) No-DAC + ASP scenarios. Air district attainment status is depicted by color with white for attainment, dashed lines for unclassified, light red for ozone nonattainment, and dark red for ozone and fine particulate matter (PM_2.5_) nonattainment. No-DAC is no disadvantaged communities, and ASP is aerated static piles.

Using the ISRM, we estimated the public health impact of air pollutants emitted from each site. In our baseline Windrow scenario, sites in the Bay Area, Southern California, and San Joaquin Valley had the highest average annual public health impact, with $7.03 × 10^6^, $4.41 × 10^6^, and $4.11 × 10^6^, respectively (Figure 4). When grouped by air district, sites in the Ventura, Bay Area, and Antelope Valley districts had the highest average annual damages with $12.91 × 10^6^, $10.37 × 10^6^, and $8.85 × 10^6^, respectively. Northern California sites had the lowest average health impact, with $0.64 × 10^6^. In total, 86% of Baseline sites were in nonattainment districts compared to 79% of No-DAC sites (Figure 4). Despite most sites being in nonattainment districts, the potential air pollution public health impact of sites ranged considerably, driven by current air quality and especially by proximity to population centers.

In our Windrow scenarios, Baseline sites caused an average of $3.55 × 10^6^ in annual damages, while No-DAC sites caused $3.28 × 10^6^ (Figures 2c and 4). The No-DAC average is slightly lower because sites in DACs were, on average, closer to population centers, where a greater number of people would be exposed to air pollutant emissions. While those living in DACs likely have less capacity to cope with the health impacts caused by air pollution, this is not directly accounted for in our analysis.^21^ Under our ASP scenarios, public health damages were reduced by 56%, with annual averages of $1.55 × 10^6^ and $1.42 × 10^6^ for the Baseline and No-DAC sites, respectively. This is due to better aeration and less frequent pile disturbance in ASP composting systems, which can reduce both NH_3_ and VOC emissions while also improving the climate benefit of composting by substantially reducing CH_4_ emissions.^49^ Using the average annual damages per site from our Windrow scenario, we estimate that all 75–100 new composting facilities would cause $266–355 million in public health damages, which is equal to 31–41% of all air pollution health damages from current electricity generation in California.^78,79^ However, if all new sites used ASP rather than windrow composting, California could reduce the total annual cost to public health from air pollutant emissions by approximately $150–200 million, or the equivalent of reducing health damages from California electricity generation by 17–23%.^78,79^

It is important to note that the climate benefit from composting is greater than the health impact caused by air pollutant emissions. Using results from our previous lifecycle assessment of SB 1383 and assuming a social cost of CO_2_, CH_4_, and N_2_O of $212 t^–1^, $2025 t^–1^, and $60,267 t^–1^, respectively, we estimate that the statewide climate benefit of SB 1383 in 2025 would be $854 million (adjusted for inflation to 2023 U.S. dollars to better compare to our public health social cost estimate).^3,80^ Additionally, we previously estimated that compost land application, as a result of SB 1383, could improve soil health across 8 × 10^5^ ha of farmland.^3^

While the flaring of captured gas at landfills emits comparatively minimal air pollutants relative to composting on a per mass of waste basis, there exists very little data on air pollutant emissions from the open face of a landfill before a gas capture system is installed.^5^ In this study, we focus on direct air pollutant emissions from composting facilities rather than on the net changes in emissions relative to landfilling. Additional research, including collection of empirical data, is needed to understand the non-combustion air pollutant emissions from landfills on a per mass of waste basis. However, using the EPA’s NH_3_ emission factor for landfilled organic waste, which is based on a single study from 1990, we estimate that NH_3_ emissions from landfilling are only 2.5–6.6% of those from composting.^5,81,82^

In our baseline Windrow scenario, transportation emissions from collecting feedstock and distributing compost to working lands accounted for just over 1% of total health damages, showing that air pollutant emissions from transportation are negligible relative to the emissions from the composting process itself. This is consistent with previous analyses, which found that greenhouse gas emissions from transportation have a negligible impact on the compost lifecycle global warming potential, especially when accounting for the avoided CH_4_ emissions from landfill diversion.^3,5^ NH_3_ emissions from composting accounted for 81% of total health damages, while composting VOC emissions were responsible for just 18%. This is because NH_3_ has a low molecular weight and a high social cost per unit mass and is a limiting factor in PM_2.5_ formation in many non-agricultural regions of the western U.S., such as Southern California.^83−85^ However, it should be noted that there is high uncertainty around modeled NH_3_ damages, especially in California, primarily due to the high seasonal and geographic variability in the ratio of NH_3_ to HNO_3_ in the atmosphere, which determines the role that NH_3_ plays in PM_2.5_ formation, and different air pollution models can produce very different results.^5,85−87^ We tested the effect of model choice on our air pollution health damages results by comparing our ISRM results with results produced using the Air Pollution Emission Experiments and Policy Version 4 (AP4) model and the Estimating Air Pollution Social Impact Using Regression (EASIUR) model.^85,88^ We found that, in our Windrow scenario, health damage results from AP4 were 59% greater than those from ISRM, while EASIUR damages were 47% less than ISRM; however, this can partly be explained by the fact that EASIUR does not account for damages from VOC emissions.

Air pollution from composting facilities not only poses a substantial public health risk but is one of the greatest barriers to siting composting facilities in California, which has some of the worst air quality in the nation.^12,18,19,84^ In order to regulate air pollutant emissions from composting, new facilities in California must go through the New Source Review permitting process, which is managed by individual air districts. While permitting rules vary between air districts and are stricter in nonattainment districts that do not meet federal air quality standards, most require the purchase of VOC emission reduction credits (ERCs), which are generated when another facility closes or reduces their emissions.^12^ However, VOC ERCs can be expensive, costing up to $127,000/t, and they may not always be available, as demand may exceed supply within a given air district.^12,89^ Most air districts have specific VOC offset emission thresholds based on the air quality in their district, and only facilities whose emissions exceed a district’s threshold are required to purchase ERCs (Table S4).^12,89^ We estimate that if a new 60 × 10^3^ t facility uses ASP rather than windrow composting, VOC emissions would fall under the threshold in 12 of 35 air districts, compared to 7 for windrows.^12^ If a facility uses ASP and reduces its throughput to 20 × 10^3^ t, it would not be required to purchase ERCs in 27 air districts.^12^ ASP composting could be a cost-saving strategy in districts with expensive ERCs, but assuming the average VOC ERC price of $18,000/t and an 80 t VOC reduction from replacing windrows with ASP, the cost savings from avoided ERC purchases would nearly equal the $1,500,000 million cost to build an ASP system for a new, average-sized facility.^49,89,90^ Other strategies for reducing air pollutant emissions, such as biochar co-composting, have also been shown to substantially reduce air pollutant emissions and could potentially be combined with ASP, but more research is needed on this topic.^91^

While VOC ERC cost is likely to play a substantial role in compost facility siting, California’s AB 617 law, which provides additional resources and protections to communities that are disproportionately impacted by air pollution, may also limit siting in some DACs.^12^ There are currently 19 AB 617 communities in the state, and through California Air Resources Board’s (CARB’s) Community Air Protection Program, these communities receive additional enforcement, air quality monitoring, and air pollution reduction planning.^92^ Composting facilities sited in these communities may have to comply with additional regulation, depending on their Community Emission Reduction Plan, and this could restrict the construction of new facilities in these vulnerable communities. While CARB is required to consider additional nominated communities annually, funding restrictions have limited the number of new AB 617 communities.^92^ An increase in the level of funding for this program could help the state ensure that new composting facilities are equitably sited. While California is currently leading the way in regulating air pollutant emissions from composting facilities, air quality concerns and siting restrictions may become an emerging issue as new organic waste diversion policies drive the expansion of composting capacity outside of California. For example, siting new facilities in New York state may be less challenging in upstate New York, which meets federal air quality standards, and more difficult in New York City and nearby counties, which are in nonattainment for ozone (see additional discussion in the Supporting Information).

### Siting Trade-Offs and Recommendations

From our analysis, we found that California has enough suitable composting sites to meet SB 1383. We also found that access to working lands is not a significant constraint for siting composting facilities in California, as only less than 1% of sites in our baseline Long Compost Transport scenario could not access a sufficient area of working lands to distribute their compost. This finding is likely unique to California and other states with a large area of working lands, but states with less evenly distributed working lands may experience a siting trade-off in access to rural land to distribute compost and access to feedstock produced primarily in population centers (see additional discussion in the Supporting Information). However, we found that access to municipal organic feedstock is an important limitation for siting new facilities in California, as about half of sites in our baseline Medium Feedstock Collection scenario could not access enough municipal feedstock to site an average-sized facility. While siting facilities closer to population centers, where organic waste is produced, improves access to feedstock, it also increases human health impacts, as more people are exposed to emissions. However, siting facilities in rural, low-population regions on the outskirts of cities may expose especially vulnerable populations to additional air pollution, as many of these communities are socioeconomically disadvantaged and already experience a high pollution burden.^36^ This problem could be mitigated through a decentralized composting system composed of a greater number of smaller, industrial-scale facilities and community-scale composting sites. In addition to likely emitting less than the ERC purchase threshold, smaller facilities would likely have greater success accessing enough municipal organic waste, as they have lower feedstock requirements and can be sited within population centers. While it remains unclear whether smaller facilities emit less air pollutant emissions on a per mass basis than larger facilities, a decentralized composting system would more equally distribute air pollution across the state and avoid concentrating health burdens in certain communities. If new sites process 20 × 10^3^ t yr^–1^, rather than 60 × 10^3^ t yr^–1^, California will need 225–300 new facilities to meet SB 1383, or only one-third to half of the suitable sites identified in this analysis. A network of community composting sites could also reduce the number of new facilities needed. While we estimated that Los Angeles County has a substantial maximum potential for community composting, a statewide analysis is needed to better understand the role that community composting could play in each of the state’s diverse regions. The unique economic challenges of operating smaller facilities and community composting projects are also unclear and should be investigated in future research.^71^

Regardless of facility size or location, we recommend the use of air pollution mitigation technology, such as ASP, to reduce health damages. The regulation of NH_3_ through an ERC program, like that used for VOCs and other air pollutants, could also reduce the net impact of siting new facilities, especially since we found that NH_3_ emissions from composting accounted for over 80% of damages. Because air quality concerns from composting are likely to become an emerging issue in nonattainment areas outside of California, we recommend that site suitability analyses conducted for other regions evaluate the potential health impacts from air pollutant emissions as we show that large composting facilities can have a substantial public health impact, especially when sited near population centers.

## Supporting Information Available

Down-selection of suitable land, suitable sites and California regions, alternative working lands proximity scenarios, alternative organic waste proximity scenarios, description of scenarios, emission factors, air pollution model comparison, cost of emission reduction credits and purchase thresholds, and siting considerations in other states (PDF)
