# Supplementary material for: Site Suitability and Air Pollution Impacts of Composting Infrastructure for California’s Organic Waste Diversion Law
Source: Environ Sci Technol. 2024 Oct 30;58(45):19913–24. doi: 10.1021/acs.est.4c06371 (PMC11562721; doi:10.1021/acs.est.4c06371)
Supplement: Supplementary file 1 — es4c06371_si_001.pdf [file es4c06371_si_001.pdf]

Supporting Information

Site Suitability and Air Pollution Impacts of Composting Infrastructure for California's Organic Waste Diversion Law

Brendan P. Harrison[1,2*], Wilson H. McNeil[2,3], Tao Dai[4,5], J. Elliott Campbell[6], Corinne D. Scown[1,2,4,5]

[1]Energy and Biosciences Institute, University of California, Berkeley, Berkeley, California 94720, United States
[2]Energy Technologies Area, Lawrence Berkeley National Laboratory, Berkeley, California 94720, United States
[3]Department of Civil and Environmental Engineering, University of California, Berkeley, Berkeley, California 94720, United States
[4]Biosciences Area, Lawrence Berkeley National Laboratory, Berkeley, California 94720, United States
[5]Life-Cycle, Economics and Agronomy Division, Joint BioEnergy Institute, Emeryville, California 94608, United States
[6]Environmental Studies Department, University of California, Santa Cruz, Santa Cruz, California 95064, United States

*Corresponding author: Brendan P. Harrison, brendanh11@berkeley.edu

Number of Pages: 11

Number of Figures: 4

Number of Tables: 4

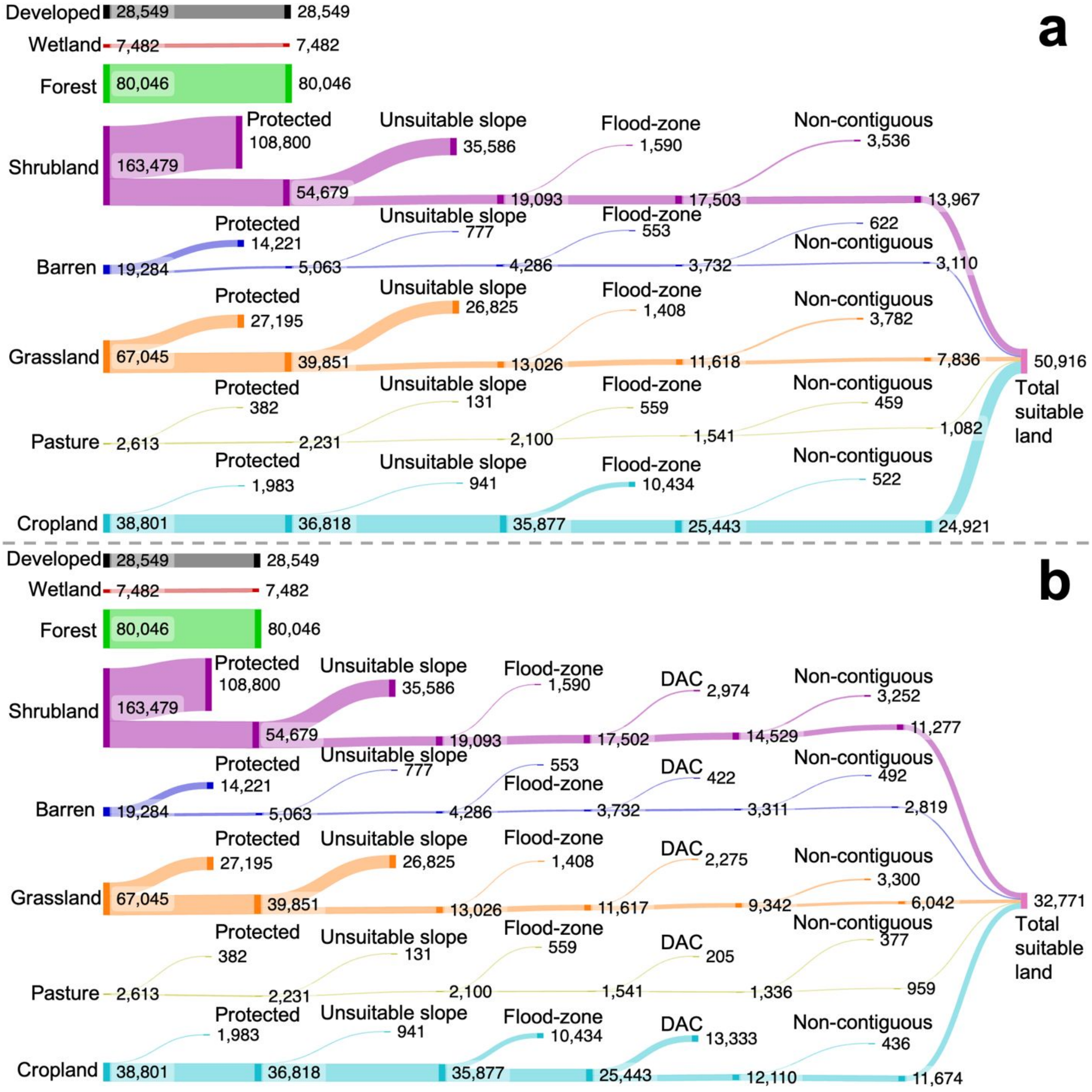

Figure S1. Sankey diagram showing down-selection of suitable land for compost facility siting for (a) baseline sites and (b) no-DAC sites. All land classified as developed, forest, and wetland are considered unsuitable. Of the five remaining land classes, additional land is excluded that is protected, has an unsuitable slope, is in a flood zone, is in a disadvantaged community (DAC) (in the case of the no-DAC scenario), and is non-contiguous (land parcels with an area less than 23.4 h) (see Methods section for further explanation). All units are in km^2.

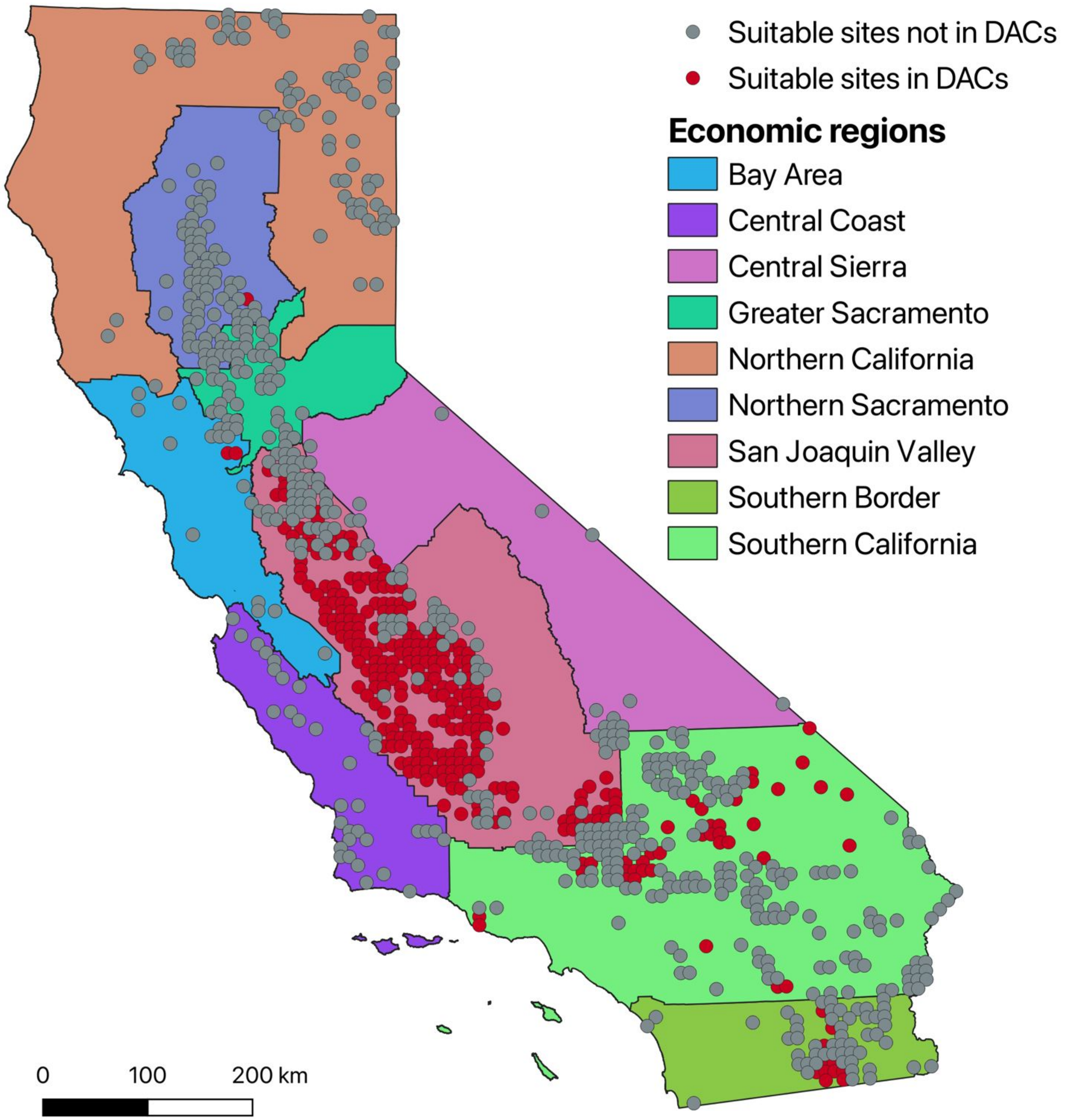

Figure S2. Suitable sites for new composting facilities across the nine economic regions of California. Gray sites are not in disadvantaged communities (DACs) and red sites are in DACs.

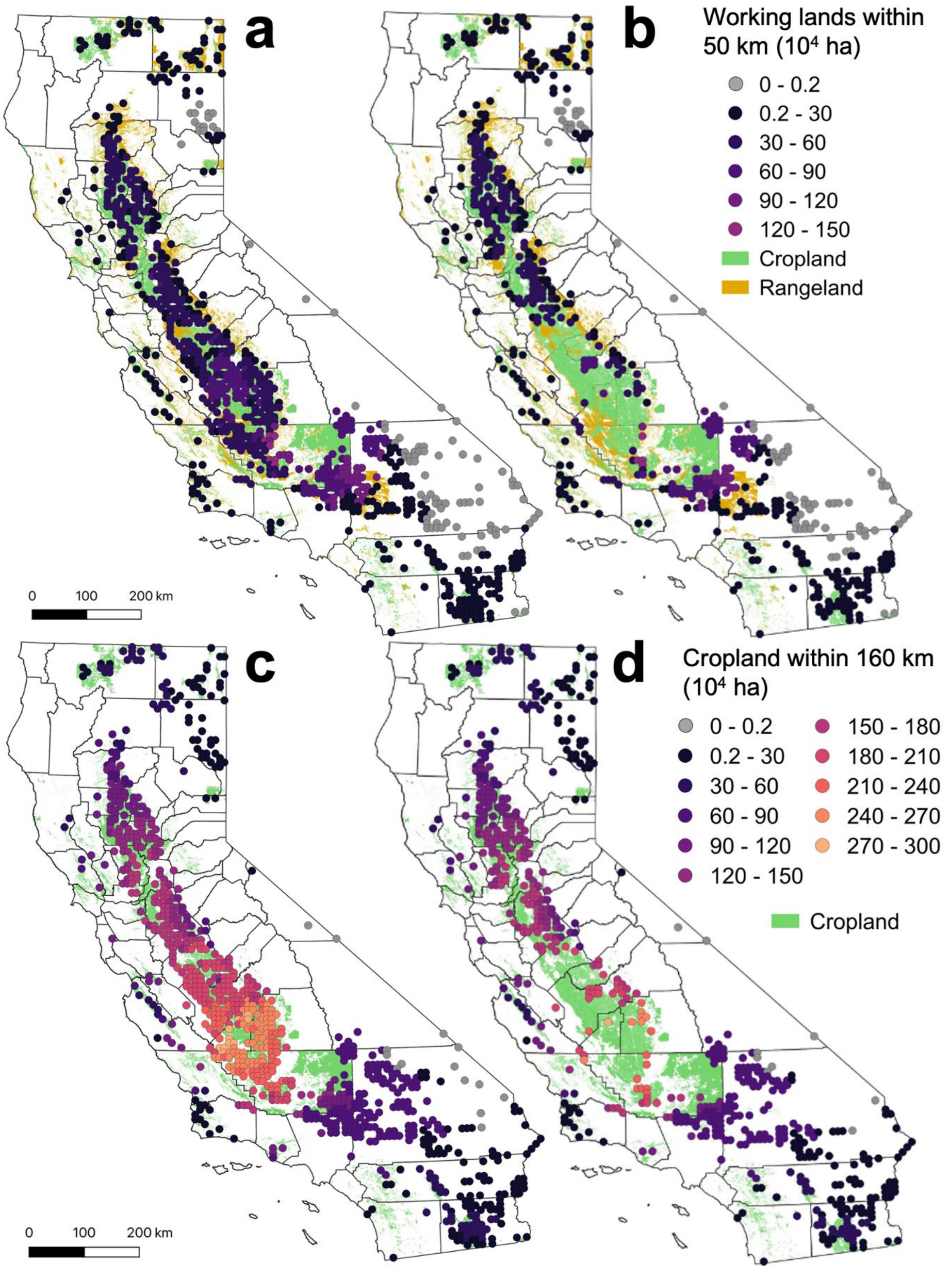

Figure S3. Area of farmland (10^4 hectares) accessible to each suitable compost facility site in the (a) Short Compost Transport, (b) No-DAC + Short Compost Transport, (c) Crop-only, and (d) No-DAC + Crop-only scenarios. Green polygons represent cropland and light brown polygons represent rangelands. No-DAC is no disadvantaged communities.

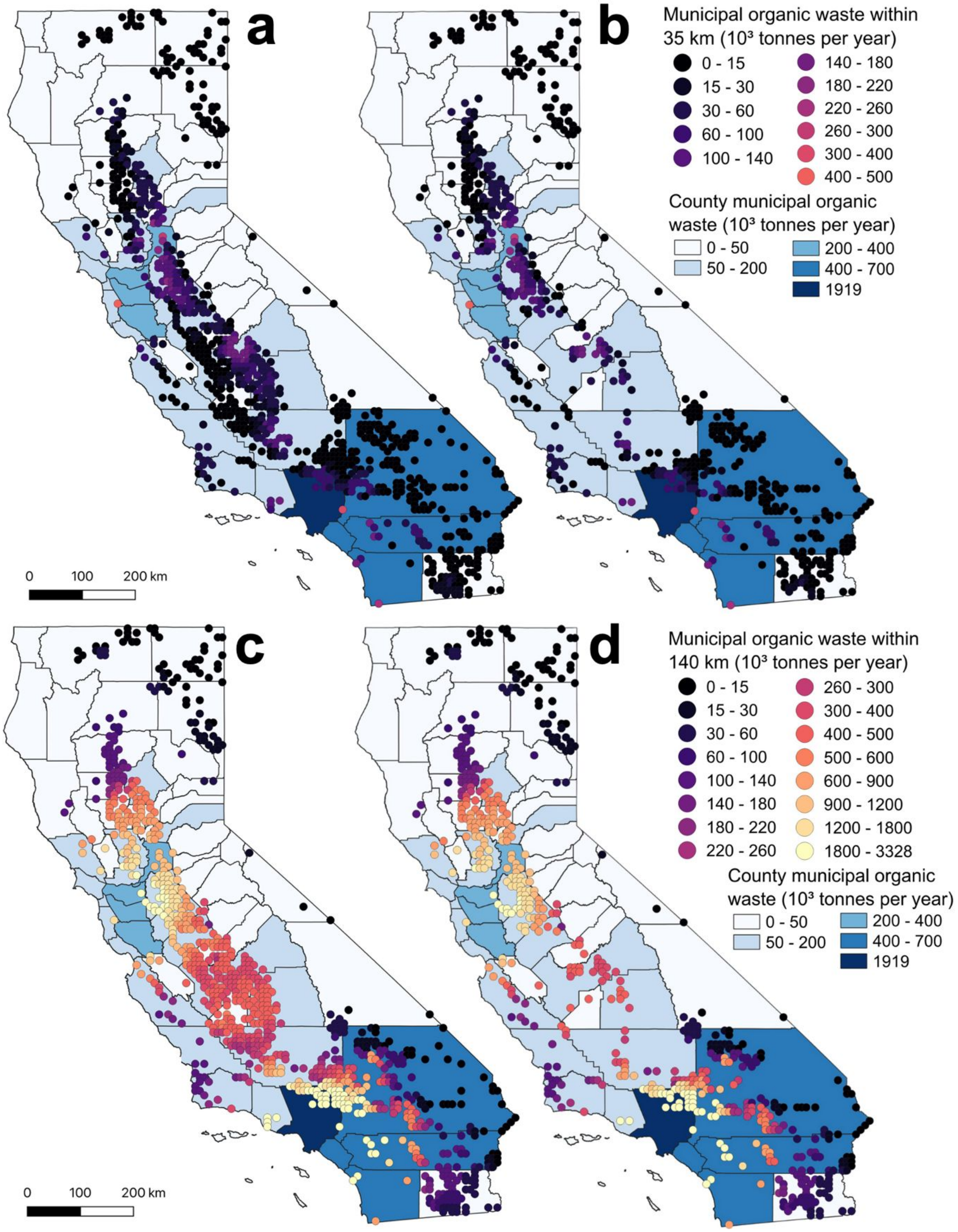

Figure S4. Amount of municipal organic waste (10^3 tonnes per year) accessible to each suitable compost facility site in the (a) Short Feedstock Collection, (b) No-DAC + Short Feedstock Collection, (c) Long Feedstock Collection, and (d) No-DAC + Long Feedstock Collection scenarios. Counties are shaded by organic waste production with darker counties producing more waste. No-DAC is no disadvantaged communities.

**Analysis**	**Scenario Name**	**Description**
Site suitability analysis	Baseline	All suitable sites included.
	No-DAC	Disadvantaged Community sites excluded.
Working Lands Proximity Analysis	Long Compost Transport	Baseline 160 km compost hauling distance.
	Short Compost Transport	50 km compost hauling distance.
	Crop-only	160 km hauling distance and rangelands excluded.
	No-DAC + Long Compost Transport	160 km hauling distance and Disadvantaged Community sites excluded.
	No-DAC + Short Compost Transport	160 km hauling distance and Disadvantaged Community sites excluded.
	No-DAC + Crop-only	160 km hauling distance, rangelands excluded, and Disadvantaged Community sites excluded.
Organic Waste Proximity Analysis	Long Feedstock Collection	140 km feedstock collection distance
	Medium Feedstock Collection	Baseline 70 km feedstock collection distance
	Short Feedstock Collection	35 km feedstock collection distance
	No-DAC + Long Feedstock Collection	140 km collection distance and Disadvantaged Community sites excluded.
	No-DAC + Medium Feedstock Collection	70 km collection distance and Disadvantaged Community sites excluded.
	No-DAC + Short Feedstock Collection	35 km collection distance and Disadvantaged Community sites excluded.
Health Impact Analysis	Windrow	Windrow composting emission factors.
	ASP	Aerated static pile composting emission factors.
	No-DAC + Windrow	Windrow composting emission factors and Disadvantaged Community sites excluded.
	No-DAC + ASP	Aerated static pile composting emission factors and Disadvantaged Community sites excluded

Table S1. Name and description of each scenario for all analyses.

	NH_3	VOC	NO_x	$PM_{2.5}$	SO_2
Windrow Composting[1] (kg/kg wet feedstock)	1.44×10^{-3}	2.53×10^{-3}	-	-	-
ASP composting[1] (kg/kg wet feedstock)	6.15×10^{-4}	1.20×10^{-3}	-	-	-
Heavy duty diesel truck[2] (g/kg fuel)	-	-	14.30	2.20	2.20×10^{-2}

Table S2. Emission factors used in health impact analysis for windrow composting, aerated static pile (ASP) composting and heavy-duty diesel trucks.

Model name	Windrow damages ($\$10^6$ yr^{-1} for average individual site)	Aerated static pile damages ($\$10^6$ yr^{-1} for average individual site)	Windrow damages ($\$10^6$ yr^{-1} for all 75-100 sites)	Aerated static pile damages ($\$10^6$ yr^{-1} for all 75-100 sites)
InMAP Source-Receptor Matrix	3.55	1.55	266-355	116-155
Air Pollution Emission Experiments and Policy Version 4	8.62	4.00	647-862	300-400
Estimating Air Pollution Social Impact Using Regression (*note: EASIUR does not account for damages from VOC emissions)*	1.89	0.85	142-189	64-85

Table S3. Comparison of air pollution health damages from our Windrow and ASP scenarios using either the InMAP Source-Receptor Matrix, the Air Pollution Emission Experiments and Policy Version 4, or the Estimating Air Pollution Social Impact Using Regression model. Results are presented for an average individual site or for the total damages from all 75-1000 new composting facilities needed to meet SB 1383, assuming average damages for each site.

Air District	Average cost of VOC ERC purchased in 2018[3] ($/metric ton)	NSR VOC ERC Purchase Threshold[4] (metric ton yr^{-1})
Amador	-	110.2
Antelope Valley	-	27.6
Bay Area	$8,451	11.0
Butte	-	110.2
Calaveras	-	110.2
Colusa	-	27.6
Eastern Kern	-	27.6
El Dorado	-	11.0
Feather River	-	11.0
Glenn	-	27.6
Great Basin	-	N/A
Imperial	$1,750	27.6
Lake County	-	27.6
Lassen	-	50.3
Mariposa	-	110.2
Mendocino	-	N/A
Modoc County	-	N/A
Mojave Desert	-	27.6
Monterey Bay	-	11.0
North Coast	-	27.6
Northern Sierra	-	N/A
No. Sonoma	-	N/A
Placer County	-	27.6
Sac. Metro	$14,937	11.0
San Diego	-	55.1
San Joaquin V.	$5,041	11.0
San Luis Obispo	$22,046	27.6
Santa Barbara	$127,000	27.6
Shasta	-	27.6
Siskiyou	-	N/A
South Coast	$21,129	4.4
Tehama	-	27.6
Tuolumne	-	N/A
Ventura	$71,871	5.5
Yolo-Solano	-	11.0

Table S4. Average cost of VOC emission reduction credits (ERC) purchased in 2018 (most recent year for which there is data)[3] and new source review (NSR) ERC purchase thresholds for each district.[4]

Potential siting challenges in other states with organic waste bans

There are currently 10 states that have adopted policies designed to reduce the disposal of organic waste in landfills (CA, CT, MA, MD, NH, NJ, NY, RI, WA, VT). A recent analysis of the effect of statewide commercial food waste bans on landfill disposal rates found that such policies have not yet reduced landfill waste, with the exception of Massachusetts'. The authors suggest that the success of Massachusetts' policy is largely due to its composting infrastructure, which is the most extensive in the country.[5] This suggests that a substantial expansion of composting infrastructure in states with organic waste bans is still needed for these policies to be successful. Here, we draw from our California site suitability analysis results to discuss potential compost distribution and air quality issues that the other nine states may experience as they expand their composting infrastructure in order to meet their policy goals. We do not discuss access to municipal organic waste feedstock because this would require a full site suitability analysis for each state, which is outside the scope of this study.

California's expansive working lands are well distributed throughout the state, and we found that nearly all candidate composting facility sites could access a sufficient area to distribute compost. This would likely also be true for New Jersey, which has a large area of farmland distributed across the western part of the state.[6] Similarly, Connecticut and Maryland have well distributed working lands and are not likely to have compost distribution issues. Rhode Island and Vermont have relatively little farmland, but they may not experience significant distribution challenges due to their low populations, especially if compost can be distributed across state lines to farmland in upstate New York, in the case of Vermont, and to farmland in eastern Connecticut or southern Massachusetts for Rhode Island. In New York, most of the state's farmland is far from New York City, home to the majority of the state's population, but compost could be distributed to ample farmland in Pennsylvania and New Jersey, if allowed. On the other hand, Washington, Massachusetts and New Hampshire have little farmland near their population centers and could experience distribution issues.[6]

While California has some of the worst air quality in the state, several other states also experience air quality issues that may impact siting decisions. For example, all counties in New Jersey and Connecticut are in nonattainment for ozone.[7] Much of Maryland is also in nonattainment, though facilities could be sited on its eastern side which does not experience air quality issues. While most of New York is in attainment for ozone, New York City, Long Island, and Westchester and Rockland counties are not, and while facilities could be sited in upstate New York, longer waste collection distances would be needed to access the waste produced in its primary population center. It should be noted that California is the only state of those with organic waste bans to have regions in nonattainment for $PM_{2.5}$.[7] Also, while we focus here on nonattainment regions that exceed federal air quality standards, additional air quality regulations at the local level could apply and be relevant for compost facility siting.

SI References

1. Nordahl, S. L., Preble, C. v., Kirchstetter, T. W., & Scown, C. D. (2023). Greenhouse gas and air pollutant emissions from composting. *Environmental Science and Technology*, *57*(6), 2235–2247. https://doi.org/10.1021/acs.est.2c05846
2. Tong, F., Jenn, A., Wolfson, D., Scown, C. D., & Auffhammer, M. (2021). Health and climate impacts from long-haul truck electrification. *Environmental Science and Technology*, *55*(13), 8514–8523. https://doi.org/10.1021/acs.est.1c01273
3. California Air Resources Board. (2020). *Emission Reduction Offset Transaction Costs Summary Report for 2018.* https://ww2.arb.ca.gov/sites/default/files/2020-05/2018_erc_report.pdf
4. Abbs, A., Reul-Chen, C. (2018). *Composting in California: Addressing Air Quality Permitting and Regulatory Issues for Expanding Infrastructure.* California Air Resources Board. https://californiacompostcoalition.org/mobius/wp-content/uploads/2022/01/CA-Compost-Comments-2018.pdf
5. Anglou, F., Sanders, R., & Stamatopoulos, I. (2024). Of the first five US states with food waste bans, Massachusetts alone has reduced landfill waste. *Science, 385*(6714), 1236-1240. DOI: 10.1126/science.adn4216
6. U.S. Department of Agriculture National Agriculture Statistics Service. (2024). *CroplandCROS*. https://croplandcros.scinet.usda.gov/
7. United States Environmental Protection Agency. (2024). *Nonattainment Areas for Criteria Pollutants (Green Book).* https://www.epa.gov/green-book/green-book-map-download
